# Contributions of Dopamine-Related Genes and Environmental Factors to Highly Sensitive Personality: A Multi-Step Neuronal System-Level Approach

**DOI:** 10.1371/journal.pone.0021636

**Published:** 2011-07-13

**Authors:** Chunhui Chen, Chuansheng Chen, Robert Moyzis, Hal Stern, Qinghua He, He Li, Jin Li, Bi Zhu, Qi Dong

**Affiliations:** 1 State Key Laboratory of Cognitive Neuroscience and Learning, Beijing Normal University, Beijing, China; 2 Department of Psychology and Social Behavior, University of California Irvine, Irvine, California, United States of America; 3 Department of Biological Chemistry and Institute of Genomics and Bioinformatics, University of California Irvine, Irvine, California, United States of America; 4 Department of Statistics, University of California Irvine, Irvine, California, United States of America; 5 Brain and Creativity Institute, University of Southern California, Los Angeles, California, United States of America; 6 Institute of Basic Research in Clinical Medicine, China Academy of Chinese Medical Sciences, Beijing, China; University of Chicago, United States of America

## Abstract

Traditional behavioral genetic studies (e.g., twin, adoption studies) have shown that human personality has moderate to high heritability, but recent molecular behavioral genetic studies have failed to identify quantitative trait loci (QTL) with consistent effects. The current study adopted a multi-step approach (ANOVA followed by multiple regression and permutation) to assess the cumulative effects of multiple QTLs. Using a system-level (dopamine system) genetic approach, we investigated a personality trait deeply rooted in the nervous system (the Highly Sensitive Personality, HSP). 480 healthy Chinese college students were given the HSP scale and genotyped for 98 representative polymorphisms in all major dopamine neurotransmitter genes. In addition, two environment factors (stressful life events and parental warmth) that have been implicated for their contributions to personality development were included to investigate their relative contributions as compared to genetic factors. In Step 1, using ANOVA, we identified 10 polymorphisms that made statistically significant contributions to HSP. In Step 2, these polymorphism's main effects and interactions were assessed using multiple regression. This model accounted for 15% of the variance of HSP (*p*<0.001). Recent stressful life events accounted for an additional 2% of the variance. Finally, permutation analyses ascertained the probability of obtaining these findings by chance to be very low, *p* ranging from 0.001 to 0.006. Dividing these loci by the subsystems of dopamine synthesis, degradation/transport, receptor and modulation, we found that the modulation and receptor subsystems made the most significant contribution to HSP. The results of this study demonstrate the utility of a multi-step neuronal system-level approach in assessing genetic contributions to individual differences in human behavior. It can potentially bridge the gap between the high heritability estimates based on traditional behavioral genetics and the lack of reproducible genetic effects observed currently from molecular genetic studies.

## Introduction

No two people are the same, with each of us having a unique and stable pattern of characteristics and behaviors. Psychologists have spent more than a century trying to understand such individual uniqueness or personality, its measurement, and its determinants (e.g., psychodynamics, social learning). Psychologists have also been interested in genetic contributions to human personality. They have traditionally used twin and adoption studies to investigate the heritability of personality. For example, traits measured by the NEO Five Factor Inventory (NEO-FFI), the Temperament and Character Inventory (TCI), as well as traits such as prosocial personality all showed moderate to high heritability, about 30–60% [1], [2], [3], [4], [5]. Similarly, personality disorders such as borderline personality disorders and DSM-IV cluster B personality disorders also showed high heritability [6], [7].

Although traditional behavioral genetic studies were able to outline the extent to which genetic factors contributed to personality, this approach was not able to unlock the “black box” of specific genes that influence variations in personality. Molecular genetic research is needed to find specific genetic polymorphisms that are related to personality. Two types of molecular genetic approaches have been used: the candidate gene approach and the genome-wide approach. For example, a recent genome-wide study found that several chromosomal regions were associated with psychoticism, extraversion, and neuroticism based on the Junior Eysenck Personality Questionnaire [8]. Another genome-wide study found that gene loci on chromosomes 1, 4, 9, 18 were related to borderline personality disorder [9]. Other studies have focused on specific candidate genes related to personality. For example, a study by Gonda and colleagues found that the presence of the short allele (*5HTTLPR* polymorphism) of the *SLC6A4* gene was significantly associated with neuroticism-related traits such as anxiety, depression, hopelessness, guilt, hostility, and aggression [10]. Similarly, other studies reported significant associations between the *5-HT(2A)* receptor gene polymorphism (*A1438G*) and self-determinism and self-transcendence [3]; between the *COMT* Val158Met polymorphism and novelty seeking [11]; and between the *DRD4* 48-bp VNTR polymorphism and novelty seeking[12], [13].

Disappointingly, however, most of the molecular genetic studies on gene-behavior connections have found only small effects of single gene loci on human traits, generally accounting for 1% or less of individual variance [5], [14], [15], far less than the heritability estimates found in behavioral-genetic studies. Moreover, many of the small effects were not consistent across studies. For example, the *COMT* Val158Met polymorphism Met allele was found to be related to high harm avoidance in some studies [16], but to low harm avoidance in others [17], [18], or had no effect [19], [20].

There are several possible explanations for this lack of reproducible findings of single gene-trait connections. Quality of samples (small sample size, ethnic stratification, age of subjects, gender composition, etc.) and differences in measures of behaviors might have contributed to the inconsistent results. A commonly proposed explanation, however, is that complex traits such as personality are likely to be polygenic, with each genetic variant making only a small contribution [14], [15]. Thus it is necessary to examine the combined effects of multiple gene loci [21], [22]. Equally important, environmental factors are likely to make direct contributions to behavioral phenotypes, or interact with genetic factors [23], [24]. Therefore, combining both genetic and environmental variance may greatly increase researchers' ability to explain individual variations in personality [25], [26]. Few genetic studies have attempted to incorporate environmental factors [27], [28], [29].

The current study adopted a multi-step neuronal system-level approach to assessing genetic and environmental contributions to personality. With this approach, we genotype multiple genes within a given neuronal system (namely the dopamine system in the present study) to quantify an individual's genetic “fingerprint” and associate the fingerprint with his/her personality traits. This approach should allow us to gauge the overall contributions of the dopamine system (based on its genetic variations) to personality, as well as to assess relative contributions of selected environmental factors. Several variations of this approach have been developed recently [30], [31], [32], [33].

The dopamine system is believed to play a major role in personality [34]. Previous research on gene-personality associations has also implicated various dopamine genes in personality variations as discussed earlier. In this study, we selected 16 dopamine-related neurotransmitter genes and tested 98 polymorphic loci that captured most variance in these genes (HapMap, www.hapmap.org). For the personality trait, we selected the high sensitivity trait as measured by the Highly Sensitive Person Scale (HSP) [35]. The HSP trait is characterized by both high levels of sensitivity to subtle stimuli and being easily over-aroused by external stimuli. This trait was selected because, as Aron [36] argued, high sensitivity is deeply rooted in the nervous system. HSP shows good psychometrical property and is significantly correlated with widely measured NEO Neuroticism [35]. For environmental factors, we selected parental warmth and exposure to stressful life events. Previous research has consistently documented the importance of parental warmth in personality development [37]. Furthermore, researchers have found that the family environment and exposure to stressful life events increase levels of sensitivity [38], [39].

## Methods

### Ethics Statement

This experiment was approved by the IRB of the State Key Laboratory of Cognitive Neuroscience and Learning at Beijing Normal University, China. A written consent form was obtained from each participant after a full explanation of the study procedure.

### Participants

480 healthy Chinese college students (mean age = 19.9 years old, standard deviation = 0.9; 208 males and 272 females) were enrolled from Beijing Normal University, Beijing, China. All were Han Chinese and in good health. Blood samples were collected for genotyping. Two participants were excluded because of poor genotyping results.

Genetic relatedness of subjects was checked following the protocol of Anderson et.al. [40] using Plink. We used 240 unrelated autosome SNPs (single nucleotide polymorphisms; r^2^<0.8) available from a larger project of the same subjects, using a threshold of 0.95 (personal communication with Dr. Anderson and Dr. Zondervan). No pair of subjects showed high relatedness (all PI_HAT smaller than or equal to 0.5).

### Behavioral measurements

*Highly Sensitive Person Scale* (HSP) [35] was used to measure participants' highly sensitive personality. It includes 27 questions about sensitivity, such as “Are you easily overwhelmed by strong sensory input?”, “Do other people's moods affect you?” and “Do you tend to be very sensitive to pain?” Participants rated each item on a 7-point scale, 1 = “Not at all” to 7 = “Extremely”. The total score of all items was used for analysis.

*Parental Warmth and Acceptance Scale* (PWAS) [41] measures perceived parental warmth with 11 items, such as “My parents really understand me” and “My parents like me the way I am; they don't try to ‘make me over’ into someone else”. Participants rated each item on a 6-point scale, 1 = “Disagree strongly” to 6 = “Agree strongly”. The total score of all items was used for analysis.

*Stressful Life Events*. This scale was adapted from similar measures used in Compas [42] and Wills, Vaccaro, and McNamara [43]. The scale has been used with cross-cultural samples including Chinese [41]. It lists 24 possible stressful events such as the death of a relative, not passing an examination, and parents getting divorced. Participants in this study had to indicate whether they experienced each event or not during early childhood (primary school years), early adolescence (secondary school years), and within the past two years (i.e., college years for this sample of college sophomores). The stressful events were counted separately for the three periods.

All scales were translated from English to Chinese by a team consisting of Chinese-English bilinguals and native English and Chinese speakers and double-checked with forward and backward translation. All scales had good reliability (Table 1) in this study. Reliability (or internal consistency) for stressful life events was not calculated because stressful life events are assumed to be relatively independent of one another, with no underlying latent factors, and only its cumulative effects are evident. English and Chinese versions of these scales are available as online supplementary materials (online supporting information S1).

**Table 1:** Means, standard deviations, Cronbach's alpha coefficients, and inter-scale correlations.

	Mean(SD)	Cronbach's alpha	Correlations			
			HSP	Parental Warmth	Stressful life events (Primary school)	Stressful life events (Secondary school)
Highly sensitive personality (HSP)	122.3(15.7)	0.817				
Parental warmth	53.0 (7.5)	0.827	−0.03			
Stressful life events (Primary school)	2.7(2.0)	—	0.09	−0.11*		
Stressful life events (Secondary school)	4.5(2.6)	—	0.12**	−0.10*	0.34**	
Stressful life events (College)	2.5(2.2)	—	0.14**	−0.18**	0.24**	0.41**

Note:

* *p*<0.05,

** *p*<0.01.

### Genetic analysis

#### Gene Selection

We selected 16 genes in four subsystems of the dopamine (DA) system: (1) dopamine synthesis (Tyrosine hydroxylase [*TH*], Dopa Decarboxylase [*DDC*]), Dopamine beta-hydroxylase [*DβH*]); (2) degradation/transport (*COMT*, *MAOA*, *MAOB*, *SLC6A3*); (3) dopamine receptor (*DRD1*, *DRD2*, *DRD3*, *DRD4*, *DRD5*); (4) dopamine modulation (4 Neurotensin genes [*NLN*, *NTS*, *NTSR1*, *NTSR2*]). These genes represent all major genes involved in these four DA subsystems in humans [44].

Dopamine synthesis involves converting the amino acid tyrosine (via Tyrosine hydroxylase [TH]) to levodopa (L-DOPA), followed by subsequent decarboxylation (by Dopa Decarboxylase [DDC]) to dopamine. Further conversion by Dopamine beta-hydroxylase (DβH) yields norepinephrine in some cells. For the degradation/transport subsystem, released dopamine is directly broken down at the synapse into inactive metabolites by two enzymes, COMT and MAO (including MAOA and MAOB). The dopamine transporter (SLC6A3), a membrane-spanning protein, pumps the neurotransmitter dopamine into the pre-synaptic neuron for reutilization. For the receptor subsystem, we include all five genes for dopamine receptors. For the modulation subsystem, we focused on Neurotensin genes, the only well characterized system that has been implicated in the modulation of dopamine signaling.

In order to sample the genetic diversity of these 16 genes we selected the tag SNPs (tSNPs) defined by the HapMap project (www.hapmap.org [phase 3], [45]), which are the minimum set of SNPs needed to sample most genetic diversity through linkage disequilibrium (LD). The tSNPs were defined by HapMap in 2007 using the four populations investigated at that time (European, African-Yoruban, Chinese, and Japanese ancestry), and used a general r^2^ value of 0.8 for identification. Additional SNPs were added for some genes because of previous finding that they were localized in regions showing evidence of strong recent natural selection ([46], [47]). These SNPs covered both coding and regulatory regions (for the latter up to l0 kb beyond the coding region). We included 25 SNPs for the DA synthesis subsystem, 23 SNPs and the *MAOA* VNTR for the DA degradation/transport subsystem, 28 SNPs and the *DRD4* VNTR for the DA receptor subsystem, and 20 SNPs for the DA modulation subsystem (online supplementary Table S1).

#### Genotyping techniques

The SNPs were genotyped using the standard Illumina GoldenGate Genotyping protocol (see Illumina GoldenGate Assay Protocol for details, www.southgene.com.cn, Shanghai South Gene Technology Co., Ltd, Shanghai, China). In addition, three genetic markers (*DRD4* VNTR, *MAOA* VNTR, and *COMT* rs4680) were ascertained by standard PCR procedures [48], [49], [50].

#### Gene data preprocessing

Two subjects with greater than 10% null genotyping results were excluded. In addition to automatic calling of genotypes, the Illumina genotyping platform supplied a quantitative quality measure known as the GenCall score. It measures how close a genotype is to the center of the cluster of other samples assigned to the same genotypes, compared with the centers of the clusters of the other genotypes. This measure ranges from 0 to 1, with a higher score indicating a more reliable result. The conventional cutoff point is .25 [51]. Of the 45,410 genotypes (95 SNPs of 478 subjects) used in the current study, 229 genotypes (0.5%) were excluded because their GenCall score was lower than .25.

Additional data cleaning included the treatment of low-frequency alleles. For SNPs with either heterozygote or minor allele homozygotes found in fewer than 10 participants (about 2%), these two genotype groups were combined. If the combined group still had fewer than 10 participants, the SNP(s) were excluded in further analysis. SNPs that showed no polymorphisms were also deleted. Hardy-Weinberg equilibrium (HWE) was calculated using the Chi square test and setting df to 1 (except for the *DRD4* VNTR; see below for details). For SNPs located on the X chromosome, only females were included in HWE calculations. Nine SNPs showed significant HW disequilibrium (*p*<0.05). These anomalous values were not the result of genotyping errors, but likely reflect the sampling biases of our college student sample, which differs from the general Chinese population sampled in HapMap. The inclusion of both tSNPs and additional SNPs in regions detected in selection screens (45,46) resulted in high LD among a number of SNPs. Eleven SNPs included in initial analysis were excluded from multiple regression analysis because of their high LD with other adjacent SNPs (*r^2^*>0.8, calculated with Plink [52]), in order to minimize “overcounting” the number of positive associations. These “redundant” SNPs showed the same or almost the same results as the linked SNPs, confirming the association. Online supplementary Table S1shows the details of all 98 polymorphic loci (96 SNPs and 2 VNTRs) included in our study: location (rs number, chromosome, position), gene, DA subsystem, allele polymorphism and frequency, Hardy Weinberg equilibrium, LD and deleted SNPs.

### Data analysis

The goal of the current study was to understand the relation between individual differences in HSP and genetic variations in the dopamine system in healthy subjects. Moving beyond the single-gene or a small number of haplotypes approaches used in typical molecular behavior genetics research, this study examined contributions of the DA system (characterized by the major genes and their associated loci) and its subsystems. Three major analyses were conducted in the present study. ANOVAs were conducted in order to detect the loci that would meet the inclusion criterion (*p*<0.05, uncorrected). Next, multiple regression analyses were conducted in order to examine the overall contribution of those SNPs (main effects and their interactions) as well as the contributions of environmental factors. Lastly, to assess the likelihood of false positives with the multiple regression approach, a series of permutation analyses were run on randomized data (by randomizing HSP scores among participants). In the following paragraphs, we describe these procedures in detail.

There are several ways to build a system-level multiple regression model with different assumptions about genetic effects (dosage vs. heterozygote-specific effects, gene-gene additive vs. interactive effects) and different methods of dealing with false positives (corrections for multiple comparisons at the SNP level, permutation, LASSO procedure) [30], [31], [32], [33]. Currently, since a system-level approach is relatively unexplored, there is not a standard procedure. Our ANOVA-regression-permutation approach has several advantages. First, it assumes theoretically that most high-level human behaviors are polygenic in nature and that each genetic variation makes small but cumulative effects on the behavior, resulting in significant contributions by a system such as the DA system [5], [11], [12], [21], [22], [34]. Multiple regression is one way to provide an estimate of system-level contributions. Second, a multiple regression procedure can detect loci with unique contributions. It can separate situations where multiple loci are significant due to LD from those where multiple loci indicate multiple association points: The former would involve one unique significant predictor, whereas the latter would involve multiple unique predictors. Third, multiple regression can easily accommodate gene-gene or SNP-SNP interactions. Fourth, to control for false positives, permutation analysis is implemented to gauge the probability of random effects at the system-level. Permutation analysis controls for Type I error at the system level (i.e., how likely one would have by chance found the same amount of contribution by the whole system to a given behavior). At the same time, it avoids Type II error by setting a conventional inclusion criterion of *p*<0.05 for any given SNP to be included in the multiple regression analysis. We believe that stringent corrections at the SNP level for system-level analysis or GWAS do not seem compatible with the theoretical perspective of polygenicity of complex human behaviors as mentioned earlier. It should be noted that, in our approach, the use of ANOVA to identify potential SNPs for regression was motivated for practical reasons. That is, ANOVA is a better screening method than multiple regression, because multiple regression is cumbersome when dealing with non-linear relationships between three genotypes of each SNP and behavior. To accommodate for potential non-linear (e.g., heterozygote advantage) effects, multiple regression has to include many pairs of dummy-coded variables that must be yoked (two dummy codes per SNP). With ANOVA as the screening step, any non-linear genetic effects can be detected and multiple regression can then accommodate non-linear effects by dummy coding relevant SNPs. Since ANOVA was used for screening for SNPs for inclusion, the criterion was set at *p*<0.05 (uncorrected) as would have been the case if multiple regression was conducted with all SNPs as potential predictors.

In this study, we built two kinds of regression models. In Model 1 (main effects), we included the loci with significant main effects based on the ANOVA results (*p*<0.05). To run multiple regression analyses, all SNPs were coded in a linear way, i.e., the major homozygote, heterozygote, minor homozygote were coded 1, 2, 3 respectively because in our results there were no cases of heterozygote advantage (see Table 2 below; no SNPs showed heterozygotes being significantly different in the same direction from both major allele homozygotes and minor allele homozygotes). In addition, we separated the data of the *DRD4* VNTR into three dummy-coded groups: “4R/4R” and, 2 repeat carriers “2R+” (e.g., 2R/2R, 2R/3R, 2R/4R, 2R/5R, 2R/6R) vs. others. This was done because 4R is the major ancestral allele and 2R is of theoretical importance among Chinese, inferred to be the result of recombination between a 4R allele and a 7R allele [53], [54]. The 2R and 7R alleles have a “blunted” response to dopamine in comparison to the ancestral 4R allele.

**Table 2:** Means and standard deviations of the HSP score, and main effects and post hoc comparisons of SNPs that showed significant main effects and used in subsequent multiple regression analysis.

SNP	Subsystem	Gene	Maj	Mean	SD	N	Het	Mean	SD	N	Min	Mean	SD	N	F	*p*	Post hoc(*p*<0.05)
rs3842748	Synthesis	*TH*	GG	121.96	15.63	447	CG	127.71	16.72	31					3.88	0.05	GG<CG
rs4929966	Synthesis	*TH*	GG	121.89	15.65	444	CG	128.12	16.10	34					4.98	0.03	GG<CG
rs1611123	Synthesis	*DBH*	GG	123.50	15.21	332	AG	119.82	16.39	129	AA	118.65	19.07	17	3.04	0.05	GG>AG
rs2975292	Degradation/Transport	*SLC6A3*	GG	121.94	15.55	371	CG	125.37	16.22	95	CC	112.09	11.20	11	4.28	0.01	GG,CG>CC
rs7131056	Receptor	*DRD2*	CC	118.77	16.48	156	AC	122.67	15.26	233	AA	127.70	14.17	89	9.54	0.00	CC<AC<AA
rs6062460	Modulation	*NTSR1*	GG	122.87	15.78	421	AG	118.40	15.06	57					4.06	0.04	GG>AG
rs12612207	Modulation	*NTSR2*	GG	123.76	15.48	214	AG	122.06	15.76	218	AA	117.00	16.06	46	3.58	0.03	GG, AG>AA
rs2561196	Modulation	*NLN*	AA	124.83	16.35	138	AG	122.11	15.45	235	GG	119.55	15.25	105	3.42	0.03	AA>GG
rs895379	Modulation	*NLN*	AA	119.05	15.63	197	AG	124.31	15.32	218	GG	125.78	15.96	63	7.73	0.00	AA<AG, GG
rs16894446	Modulation	*NLN*	GG	124.78	15.77	188	AG	121.43	15.71	223	AA	118.49	14.91	67	4.71	0.01	GG>AG, AA

Nine typed loci are on the x-chromosome (5 SNPs on *MAOA*, 1 *MAOA*_VNTR, 3 SNPs on *MAOB*), resulting in two genetic groups for males and three groups for females. We conducted ANOVA and post hoc tests (when there were at least five cases per cell) to determine the best way to condense the females into two groups. ANOVA results showed that only rs929095 had a marginal main effect on HSP (*p* = 0.052), which was due to a significant difference between major allele homozygotes and heterozygotes. This indicated that combining heterozygotes with minor allele homozygotes would create equivalent groups for the two sexes without missing significant findings. Thus SNPs of *MAOA* and *MAOB* were coded as 1 (major allele homozygotes) and 2 (others).

In Model 2, we added interaction terms among those variables included in Model 1. Codes of these SNPs were de-meaned first, and multiplications of de-meaned codes of every pair of SNPs were used as the interaction terms. Forward stepwise regression was used to search for significant interaction effects among the large number of potential interactions. Model comparisons were made to ascertain the significance of adding interaction terms.

Finally, permutation analyses were conducted to assess the likelihood of obtaining our results under different assumptions. Basic multiple linear regressions assume linearity, normality, independence (or non-collinearity) among predictors, non-correlated errors, etc. Because these criteria are difficult to meet, the probability of significance we obtained for our results may be too liberal. To derive more stringent criteria, we did permutation analyses. We kept the genetic structure intact and randomized behavior data (HSP), then repeated the above process on the randomized data. Specifically, for each permutation, we 1) used all 98 loci to run ANOVAs on the randomized HSP data, 2) selected SNPs with significant effects (*p*<0.05, the number of significant SNPs varied across permutations) and 3) used the selected SNPs in the regression models with these loci (Model 1) or loci plus their interactions using the forward stepwise regression (Model 2). Permutation was done 1000 times to yield a distribution of R^2^. Based on that distribution, the probability of obtaining the observed R^2^ was determined.

We estimated the unique contribution of environmental factors by adding them into the above two models using the forward stepwise procedure. Any variable entered in the model meant its contribution to HSP was not accounted for by genetic information. Monte Carlo simulation/permutation and model comparisons were run again for these models.

## Results

Table 1 shows the mean total score, standard deviations, reliability estimates, and inter-correlations among the self-report measures. Highly sensitive personality was not correlated with parental warmth, but was significantly positively correlated with the number of stressful life events during secondary school and college years. Parental warmth was negatively correlated with the number of stressful life events. Stressful life events in three periods were positively correlated with one another. Finally, there was no significant gender difference in HSP, t(476) = −1.2, *p* = 0.23.

Of the 87 polymorphisms that survived LD testing (see Methods), 10 showed main effects with *p*<0.05. Individuals who were major allele homozygotes for rs16894446 or rs1611123, heterozygotes for rs4929966 or rs3842748, or minor allele homozygotes for rs7131056 reported higher sensitivity on the HSP scale, whereas individuals who were major allele homozygotes for rs7131056 or rs895379, heterozygotes for rs6062460, or minor allele homozygotes for rs2975292 or rs12612207 or rs2561196 reported less sensitivity (Table 2, and online supplementary Table S2 for detailed information of all 98 loci).

These 10 SNPs were used in regression analysis to build Model 1 (the main effects model). Table 3 shows the results of the multiple regression analysis. The model accounted for 12% (10% adjusted) of the variance of HSP, F (10, 466) = 6.18, *p* = 7*10^−9^. We then added in two-way interaction terms of these SNPs using the forward stepwise procedure. For the 10 SNPs showing significant main effects, there were 45 potential interactions, and 3 of them entered the final model. The R^2^ increased to 0.15 and adjusted R^2^ to 0.13, F (13, 463) = 6.37, *p* = 4*10^−11^. To specify the contributions of the four DA subsystems, we further estimated the effects of each subsystem. Model 1 R^2^ of the synthesis, degradation/transport, receptor, and modulation subsystems was 0.02, 0.00, 0.04, and 0.06, respectively. The corresponding adjusted R^2^ were 0.01, 0.00, 0.03, and 0.05, respectively. Because all significant interaction terms were across subsystems, the estimates for Model 2 at the subsystem level were the same as Model 1.

**Table 3:** Two regression models for HSP with genetic data only.

			Model 1			Model 2		
Regressor	Gene 1	Gene 2	B	T	*p*	B	T	*p*
rs12612207	*NTSR2*		−2.79	−2.63	0.01	−2.75	−2.62	0.01
rs2975292	*SLC6A3*		0.88	0.62	0.54	0.96	0.68	0.50
rs2561196	*NLN*		2.90	1.57	0.12	2.95	1.62	0.11
rs895379	*NLN*		3.98	2.68	0.01	4.19	2.85	0.00
rs16894446	*NLN*		−3.14	−2.02	0.04	−3.34	−2.18	0.03
rs1611123	*DBH*		−2.88	−2.27	0.02	−3.35	−2.67	0.01
rs3842748	*TH*		−0.37	−0.07	0.94	1.19	0.22	0.82
rs4929966	*TH*		6.77	1.31	0.19	6.39	1.26	0.21
rs7131056	*DRD2*		4.57	4.63	0.00	4.20	4.31	0.00
rs6062460	*NTSR1*		−5.36	−2.52	0.01	−4.71	−2.25	0.03
rs12612207-rs2975292	*NTSR2*	*SLC6A3*				−6.77	−3.27	0.00
rs2975292 -rs2561196	*SLC6A3*	*NLN*				−4.95	−2.38	0.02
rs3842748 -rs7131056	*TH*	*DRD2*				8.18	2.10	0.04

Note: ‘Gene 1’ and ‘Gene 2’ are the corresponding genes for each SNP; ‘B’ is the regression coefficient, ‘T’ and ‘*p*’ are t-test results.

**Figure 1:**
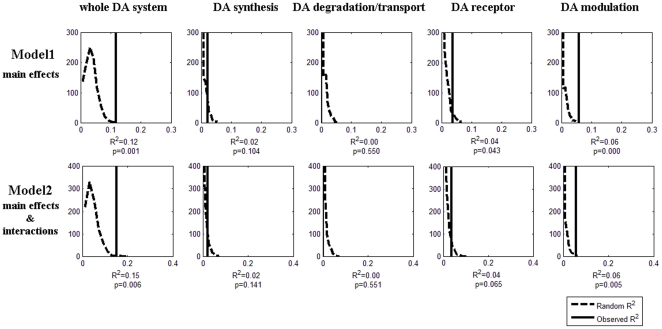
Permutation results for the two genetic models: Model 1 (first row) and Model 2 (second row); for the whole DA system, and the synthesis, degradation/transport, receptor, and modulation subsystems respectively. The dashed line represents empirical distribution of R^2^ obtained from the randomized data, and the solid vertical line represents R^2^ obtained from the actual data.

To validate these results, Monte Carlo permutation was conducted. Figure 1 shows the permutation results of R^2^: first row for Model 1, and second row for Model 2. The five columns were for the whole DA system, the DA synthesis subsystem, the DA degradation/transport subsystem, the DA receptor subsystem, and the DA modulation subsystem. In each subplot, the X axis represents R^2^, and the Y axis represents the number of occurrences of a given R^2^ in 1000 permutations. The curve represents the distribution of R^2^ based on the permutated data, whereas the vertical line indicates actual R^2^ obtained in this study. Based on these permutations, the probability of obtaining the R^2^ found in Model 1 was 0.001 (the whole model), 0.104 (synthesis), 0.550 (degradation/transport), 0.043(receptors), and <0.001 (modulation). The corresponding probabilities for Model 2 were 0.006, 0.141, 0.551, 0.065, and 0.005. Permutation of adjusted R^2^ showed the same pattern of significance. These results indicate that the DA system, especially its modulation and receptor subsystems, contributes substantially to HSP.

**Figure 2:**
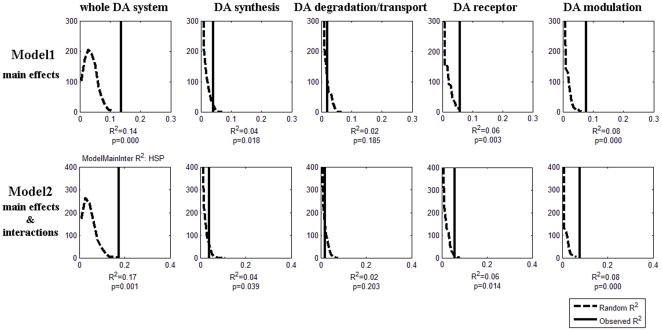
Permutation results for the two models including both genetic and environmental factors. Presented in the same manner as Figure 1.

To test if environmental factors (parental warmth and stressful life events) made unique contributions to individual differences in sensitivity, we added these variables into the regression models using the forward stepwise method. Stressful life events during college (i.e., recent stressful life events) was a significant predictor of HSP. This variable accounted for about 2% additional variance in HSP. Total R^2^ increased to 0.14 (permutation *p*<0 .001) for Model 1 and 0.17 (permutation *p* = 0.001) for Model 2, and adjusted R^2^ increased to 0.11 (permutation *p*<0.001) and 0.15 (permutation *p* = 0.004), respectively (see Figure 2 and Table 4).

**Table 4:** Two regression models for HSP with both genetic data and environmental variables.

			Model 1			Model 2		
Regressor	Gene 1	Gene 2	B	T	*p*	B	T	*p*
rs12612207	*NTSR2*		−2.72	−2.59	0.01	−2.88	−2.78	0.01
rs2975292	*SLC6A3*		0.92	0.65	0.52	1.22	0.87	0.39
rs2561196	*NLN*		3.03	1.66	0.10	2.85	1.59	0.11
rs895379	*NLN*		4.17	2.83	0.00	4.13	2.85	0.00
rs16894446	*NLN*		−3.15	−2.04	0.04	−3.33	−2.20	0.03
rs1611123	*DBH*		−2.88	−2.30	0.02	−3.46	−2.79	0.01
rs3842748	*TH*		−0.51	−0.10	0.92	0.26	0.05	0.96
rs4929966	*TH*		6.52	1.28	0.20	6.37	1.27	0.21
rs7131056	*DRD2*		4.63	4.75	0.00	4.22	4.38	0.00
rs6062460	*NTSR1*		−5.26	−2.50	0.01	−4.70	−2.27	0.02
rs12612207-rs2975292	*NTSR2*	*SLC6A3*				−6.76	−3.30	0.00
rs12612207-rs1611123	*NTSR2*	*DBH*				4.04	2.18	0.03
rs2975292 -rs2561196	*SLC6A3*	*NLN*				−5.86	−2.85	0.00
Stressful life events (College)			1.03	3.28	0.00	1.15	3.72	0.00

Note: See explanations of the terms in Table 3.

Model comparisons showed that models with gene-gene interactions or environment factors fit the data better than those without, as indicated by smaller AIC and BIC values (Table 5).

**Table 5:** Comparison of regression models.

	models	R^2^	ΔR^2^	−2LL	df	*p*	AIC	BIC
Genetic data only	Model 1	0.12	-	3921	10	-	3941	3983
	Model 2	0.15	0.03	3902	13	2.7*10^−4^	3928	3982
Genetic data and environment factors	Model 1	0.14	0.02	3910	11	9.1*10^−4^	3932	3978
	Model 2	0.17	0.05	3889	14	1.9*10^−6^	3917	3976

Note: R^2^ is the proportion of variance explained by the model; ΔR^2^ is the difference in R^2^ between the current model and first model; −2LL is the log likelihood of the regression model multiplied by −2; *p* was calculated to estimate change in −2LL by Chi-square distribution with df that equals the difference of dfs between models. AIC (Akaike's information criterion) and the BIC (Bayesian information criterion) are information-theory measures of goodness of model fit.

## Discussion

By using a multi-step neuronal system-level approach, the current study was able to assess the contribution of the dopamine system to the highly sensitive personality trait. We tested 98 polymorphisms related to the dopamine system, and identified 10 loci on seven genes that were related to highly sensitive personality. We then examined their cumulative effects (main and interaction effects) using regression models. Results showed that the DA system accounted for about 15% of the variance of HSP. This estimate, based on the assumption of polygenicity and gene-gene interactions, brings the observed total genetic contribution to the variability of a personality trait much closer to the estimated genetic contribution based on traditional behavioral genetics studies (30%–60%). The idea of adding or multiplying the effects of polymorphisms to better understand the genetic basis of human traits has been adopted by other researchers. Most recently, estimates of multiple gene loci's contributions to height [32] and sensation-seeking personality [33] have been reported. The current study found that genes within the dopamine system make a significant contribution to the variability observed in the highly sensitive personality trait.

Our results also indicate that genes of different subsystems of the dopamine system were related to personality. Given the complexity of the dopamine system, it makes intuitive sense that variations in different subsystems (such as synthesis, degradation/transport, receptors, modulation) would all contribute to individual differences in behavior. Single-gene approaches, then, without considering (or statistically controlling for) other subsystems, would likely yield mixed findings. With the multi-step approach used in this study, we were able to identify multiple genes and determine which genes were likely to make unique contributions. Polymorphisms in *TH*, *DβH*, *SLC6A3*, *DRD2*, *NLN*, *NTSR1*, *NTSR2* were identified as associated with HSP. Tyrosine hydroxylase (TH) catalysizes tyrosine to levodopa (L-DOPA); DβH converts dopamine to norepinephrine; SLC6A3 is a dopamine transporter; DRD2 is a dopamine receptor; and NLN, NTSR1, and NTSR2 belong to the neurotensin system, proteins reported to be structurally co-localized with and to functionally interact with the dopamine system, especially with DRD2 [55], [56], [57]. Indeed, previous studies have reported evidence of involvement of several of these genes in personality or related measures. For example, the *TH* gene was reported to be related to essential hypertension in Chinese subjects [58], [59]. The *DRD4* and *DRD2* genes were reported to be associated with childhood temperament, extraversion, and antisocial behavior [5], [60], [61]. It is possible that these traits are related to highly sensitive personality. Neurotensin has been reported to be associated with schizophrenia [55], [62], [63] and memory consolidation [64], which may also be mediated by high sensitivity. Given that most of these newly identified SNPs related to HSP have unknown function, future work needs to examine their underlying biochemical impact. As discussed above, studies that relied on single gene-behavior association might not yield consistent results perhaps due to the small effects of each gene. We believe that system-level results should be more replicable than those from single-gene or single-polymorphism studies.

Another contribution of our approach is a systematic examination of gene-gene interactions. We found that only interactions between subsystems made unique contribution to HSP, indicating that these four subsystems do not function independently, but cumulatively and interactively. These results are consistent with theoretical models that assume interactions among these subsystems. Further research is needed to delineate the precise processes of such interactions.

Our results further showed that environmental factors such as stressful life events accounted for unique variations of the personality trait. Our aim in this study was to show that, with our multi-step approach, genetic and environmental contributions can be assessed in the same model. It was not meant to exhaust all possible environmental factors that may impact the highly sensitive personality trait. We found that recent stressful life events made unique contributions. The effects of earlier stressful life events and parental warmth were absorbed by their covariance with recent life events.

There are several limitations to the current study. First of all, we only selected 98 polymorphisms related to the major genes in the dopamine system. While these polymorphisms were chosen to capture much of the genetic diversity of these genes via LD, some critical polymorphisms could have been missed. The DA system was chosen based on previous empirical evidence and theoretical arguments for the importance of this system in personality. However, other neuronal systems such as the serotonin system may also contribute to personality [3], [10]. Such systems should be examined separately or in conjunction with the DA system. Second, we used two environmental factors to demonstrate the possibility of incorporating them into our model. A careful selection of other environmental factors or other measures of parenting and social environments would help refine our model. Future research should also incorporate other examples of gene-environment interactions [23], [24], [25], [65], [66], [67], [68], [69]. This is theoretically and computationally difficult to do with a large number of SNPs, but should be feasible after a small set of specific SNPs have been replicated. Lastly, although the English versions of the questionnaires are widely used in research, our Chinese versions need further validation.

To summarize, the current study adopted a new approach to study gene-behavior associations that considers the polygenic nature of behavior and examines gene-gene interactions and incorporates both genetic and environmental factors, often ignored in prior studies [70]. This approach characterizes individuals by their genetic diversity at a neuronal system level and assesses the overall contribution of that system to a given behavior. It has the potential to bridge the gap between the high estimates of the genetic contributions to variability obtained from heritability studies and the low estimates currently observed for individual genes in molecular genetic studies.

## Supporting Information

Table S1 Detailed information of the loci used in this study.
(DOC)

Table S2 Means and standard deviations of HSP score for each polymorphism, and main effects and post hoc comparisons of each locus. Rows in bold style were those that showed significant main effects and were used in subsequent multiple regression analysis.
(DOC)

Supporting Information S1 English and Chinese versions of scales used in this study.
(DOC)
