# Supplementary material for: Contributions of Dopamine-Related Genes and Environmental Factors to Highly Sensitive Personality: A Multi-Step Neuronal System-Level Approach
Source: PLoS One. 2011 Jul 13;6(7):e21636. doi: 10.1371/journal.pone.0021636 (PMC3135587; doi:10.1371/journal.pone.0021636)
Supplement: Table S1 — Detailed information of the loci used in this study. (DOC) [file pone.0021636.s001.doc]

Table S1. Detailed information of the loci used in this study

| SNP | Chr | Position | Gene | Subsystem | Maj | N | Het | N | Min | N | Miss | HWE  p | LD  r^2^>0.8^ξ^ | deleted^ξ^ |
| --- | --- | --- | --- | --- | --- | --- | --- | --- | --- | --- | --- | --- | --- | --- |
| rs3842748 | 11 | 2137971 | TH | Synthesis | GG | 447 | CG | 30 | CC | 1 | 0 | 0.51 |  |  |
| rs2070762 | 11 | 2142911 | TH |  | AA | 175 | AG | 219 | GG | 81 | 3 | 0.38 |  |  |
| rs6356 | 11 | 2147527 | TH |  | AA | 325 | AG | 132 | GG | 21 | 0 | 0.11 |  |  |
| rs4930046 | 11 | 2153724 | TH |  | AA | 368 | AG | 88 | GG | 21 | 1 | <0.01 |  |  |
| rs4929966 | 11 | 2154012 | TH |  | GG | 444 | CG | 32 | CC | 2 | 0 | 0.10 |  |  |
| rs11238131 | 7 | 50506083 | DDC |  | AA | 131 | AG | 243 | GG | 103 | 1 | 0.62 |  |  |
| rs11238133 | 7 | 50510408 | DDC |  | AA | 132 | AC | 224 | CC | 121 | 1 | 0.19 | 1 | Y |
| rs11238134 | 7 | 50510421 | DDC |  | AA | 133 | AC | 223 | CC | 121 | 1 | 0.16 | 1 |  |
| rs3887825 | 7 | 50512587 | DDC |  | AA | 174 | AG | 221 | GG | 83 | 0 | 0.38 |  |  |
| rs3807566 | 7 | 50531698 | DDC |  | CC | 217 | AC | 204 | AA | 57 | 0 | 0.40 |  |  |
| rs1817074 | 7 | 50541506 | DDC |  | AA | 317 | AG | 141 | GG | 20 | 0 | 0.39 | 2 |  |
| rs7808025 | 7 | 50544397 | DDC |  | GG | 316 | AG | 143 | AA | 19 | 0 | 0.58 | 2 | Y |
| rs7786398 | 7 | 50580400 | DDC |  | GG | 141 | AG | 224 | AA | 113 | 0 | 0.19 | 3 |  |
| rs10499695 | 7 | 50586098 | DDC |  | AA | 158 | AG | 225 | GG | 95 | 0 | 0.36 | 3 | Y |
| rs6962356 | 7 | 50589435 | DDC |  | AA | 94 | AG | 359 | GG | 24 | 1 | <0.01 |  |  |
| rs6969081 | 7 | 50591999 | DDC |  | TT | 155 | AT | 233 | AA | 90 | 0 | 0.88 | 3 |  |
| rs2007153 | 9 | 135493640 | DBH |  | GG | 203 | AG | 204 | AA | 71 | 0 | 0.10 |  |  |
| rs1108580 | 9 | 135494935 | DBH |  | AA | 328 | AG | 134 | GG | 16 | 0 | 0.61 |  |  |
| rs1611123 | 9 | 135498904 | DBH |  | GG | 332 | AG | 129 | AA | 17 | 0 | 0.32 |  |  |
| rs1541332 | 9 | 135501337 | DBH |  | AA | 447 | AG | 30 | GG | 1 | 0 | 0.51 |  |  |
| rs2519154 | 9 | 135502096 | DBH |  | GG | 369 | AG | 102 | AA | 6 | 1 | 0.72 |  |  |
| rs6479643 | 9 | 135504489 | DBH |  | GG | 242 | CG | 198 | CC | 37 | 1 | 0.69 |  |  |
| rs77905 | 9 | 135507918 | DBH |  | GG | 395 | AG | 76 | AA | 7 | 0 | 0.14 |  |  |
| rs732833 | 9 | 135510483 | DBH |  | GG | 145 | AG | 235 | AA | 98 | 0 | 0.88 |  |  |
| rs2073837 | 9 | 135512749 | DBH |  | GG | 136 | AG | 231 | AA | 111 | 0 | 0.50 |  |  |
| rs737866 | 22 | 18310109 | COMT | Degradation/Transport | AA | 246 | AG | 197 | GG | 35 | 0 | 0.60 |  |  |
| rs5993883 | 22 | 18317638 | COMT |  | AA | 172 | AC | 234 | CC | 72 | 0 | 0.60 |  |  |
| rs740603 | 22 | 18325177 | COMT |  | AA | 153 | AG | 240 | GG | 71 | 14 | 0.14 |  |  |
| rs2239393 | 22 | 18330428 | COMT |  | AA | 198 | AG | 218 | GG | 62 | 0 | 0.87 | 4 | Y |
| rs4680 | 22 | 18331271 | COMT |  | VV | 256 | VM | 178 | MM | 30 | 14 | 0.90 |  |  |
| rs4646316 | 22 | 18332132 | COMT |  | GG | 209 | AG | 210 | AA | 58 | 1 | 0.64 | 4 |  |
| rs165774 | 22 | 18332561 | COMT |  | GG | 369 | AG | 103 | AA | 6 | 0 | 0.69 |  |  |
| rs929095 | X | 43247894 | MAOA |  | GG | 196 | CG | 128 | CC | 154 | 0 | 0.48 |  |  |
| rs1181286 | X | 43300472 | MAOA |  | CC | 396 | AC | 53 | AA | 29 | 0 | 0.52 | 5 |  |
| rs1181289 | X | 43325208 | MAOA |  | TT | 396 | AT | 52 | AA | 30 | 0 | 0.95 | 5 | Y |
| rs5906974 | X | 43436265 | MAOA |  | GG | 217 | AG | 140 | AA | 121 | 0 | 0.13 | 6 | Y |
| rs909525 | X | 43438146 | MAOA |  | GG | 217 | AG | 140 | AA | 120 | 1 | 0.13 | 6 |  |
| MAOA_VNTR | X |  | MAOA |  | VV | 210 | VM | 130 | MM | 129 | 9 | 0.61 |  |  |
| rs1799836 | X | 43512943 | MAOB |  | AA | 352 | AG | 77 | GG | 49 | 0 | 0.22 |  |  |
| rs6651806 | X | 43573908 | MAOB |  | AA | 381 | AC | 68 | CC | 29 | 0 | 0.71 |  |  |
| rs5905512 | X | 43611338 | MAOB |  | AA | 284 | AG | 117 | GG | 77 | 0 | 0.17 |  |  |
| rs1042098 | 5 | 1447815 | SLC6A3 |  | AA | 383 | AG | 89 | GG | 5 | 1 | 0.95 |  |  |
| rs40184 | 5 | 1448077 | SLC6A3 |  | GG | 260 | AG | 188 | AA | 30 | 0 | 0.61 |  |  |
| rs6347 | 5 | 1464412 | SLC6A3 |  | AA | 370 | AG | 99 | GG | 9 | 0 | 0.43 |  |  |
| rs37022 | 5 | 1468629 | SLC6A3 |  | AA | 120 | AT | 239 | TT | 119 | 0 | 1.00 |  |  |
| rs2975292 | 5 | 1472932 | SLC6A3 |  | GG | 371 | CG | 95 | CC | 11 | 1 | 0.10 | 7 |  |
| rs10053602 | 5 | 1481135 | SLC6A3 |  | AA | 373 | AG | 93 | GG | 12 | 0 | 0.04 | 7 | Y |
| rs403636 | 5 | 1491354 | SLC6A3 |  | CC | 222 | AC | 204 | AA | 52 | 0 | 0.62 |  |  |
| rs2937639 | 5 | 1496728 | SLC6A3 |  | AA | 350 | AG | 114 | GG | 14 | 0 | 0.21 |  |  |
| rs686 | 5 | 174801306 | DRD1 | Receptor | AA | 351 | AG | 114 | GG | 13 | 0 | 0.31 | 8 | Y |
| rs1799914 | 5 | 174802511 | DRD1 |  | GG | 442 | AG | 35 | AA | 1 | 0 | 0.73 |  |  |
| rs4532 | 5 | 174802756 | DRD1 |  | AA | 351 | AG | 114 | GG | 12 | 1 | 0.46 | 8 | Y |
| rs5326 | 5 | 174802802 | DRD1 |  | GG | 296 | AG | 157 | AA | 23 | 2 | 0.71 |  |  |
| rs265981 | 5 | 174803508 | DRD1 |  | GG | 356 | AG | 110 | AA | 12 | 0 | 0.32 | 8 |  |
| rs6277 | 11 | 112788669 | DRD2 |  | GG | 408 | AG | 69 | AA | 1 | 0 | 0.28 | 9 | Y |
| rs2734839 | 11 | 112791700 | DRD2 |  | GG | 398 | AG | 63 | AA | 1 | 16 | 0.36 | 9 |  |
| rs17529477 | 11 | 112822277 | DRD2 |  | GG | 435 | AG | 41 | AA | 2 | 0 | 0.34 |  |  |
| rs17601612 | 11 | 112822955 | DRD2 |  | GG | 441 | CG | 36 | CC | 1 | 0 | 0.77 |  |  |
| rs4245147 | 11 | 112823217 | DRD2 |  | AA | 322 | AG | 139 | GG | 17 | 0 | 0.68 |  |  |
| rs7131056 | 11 | 112834984 | DRD2 |  | CC | 156 | AC | 233 | AA | 89 | 0 | 0.90 |  |  |
| rs2134655 | 3 | 115340891 | DRD3 |  | GG | 252 | AG | 198 | AA | 27 | 1 | 0.14 |  |  |
| rs324035 | 3 | 115351544 | DRD3 |  | CC | 343 | AC | 120 | AA | 15 | 0 | 0.26 |  |  |
| rs10934256 | 3 | 115368342 | DRD3 |  | CC | 256 | AC | 196 | AA | 25 | 1 | 0.11 |  |  |
| rs7638961 | 3 | 115377084 | DRD3 |  | AA | 228 | AG | 212 | GG | 38 | 0 | 0.24 |  |  |
| DRD4VNTR | 11 | 144500 | DRD4 |  | 4/4 | 279 | 2/+ | 140 | other | 57 | 2 | －* |  |  |
| rs7933153 | 11 | 623647 | DRD4 |  | CC | 452 | AC | 26 | AA | 0 | 0 | 0.54 |  |  |
| rs12720366 | 11 | 626398 | DRD4 |  | AA | 247 | AG | 191 | GG | 40 | 0 | 0.72 |  |  |
| rs12720424 | 11 | 631192 | DRD4 |  | AA | 268 | AC | 181 | CC | 29 | 0 | 0.83 |  |  |
| rs11604855 | 11 | 631991 | DRD4 |  | GG | 306 | AG | 155 | AA | 17 | 0 | 0.63 |  |  |
| rs12233771 | 4 | 9162063 | DRD5 |  | TT | 120 | AT | 247 | AA | 111 | 0 | 0.46 |  |  |
| rs9884669 | 4 | 9172392 | DRD5 |  | CC | 228 | AC | 214 | AA | 36 | 0 | 0.14 |  |  |
| rs4102942 | 4 | 9177576 | DRD5 |  | GG | 105 | AG | 325 | AA | 47 | 1 | <0.01 |  |  |
| rs7655090 | 4 | 9374973 | DRD5 |  | AA | 133 | AG | 239 | GG | 106 | 0 | 0.94 |  |  |
| rs10033951 | 4 | 9388678 | DRD5 |  | GG | 92 | AG | 328 | AA | 58 | 0 | <0.01 |  |  |
| rs2867383 | 4 | 9397033 | DRD5 |  | AA | 145 | AG | 232 | GG | 101 | 0 | 0.65 |  |  |
| rs1850744 | 4 | 9399810 | DRD5 |  | GG | 303 | AG | 174 | AA | 0 | 1 | <0.01 |  |  |
| rs7685396 | 4 | 9403822 | DRD5 |  | CC | 319 | AC | 150 | AA | 2 | 7 | <0.01 |  |  |
| rs4697695 | 4 | 9524948 | DRD5 |  | GG | 276 | AG | 173 | AA | 26 | 3 | 0.87 |  |  |
| rs2405254 | 12 | 84541750 | NTS | Modulation | TT | 138 | AT | 216 | AA | 124 | 0 | 0.04 |  |  |
| rs12313658 | 12 | 84795194 | NTS |  | GG | 438 | AG | 39 | AA | 1 | 0 | 0.89 |  |  |
| rs1024076 | 12 | 85028825 | NTS |  | GG | 222 | AG | 210 | AA | 46 | 0 | 0.72 |  |  |
| rs10506933 | 12 | 85165682 | NTS |  | CC | 198 | CG | 223 | GG | 57 | 0 | 0.63 |  |  |
| rs2427399 | 20 | 60801890 | NTSR1 |  | GG | 287 | AG | 167 | AA | 24 | 0 | 0.96 |  |  |
| rs6062460 | 20 | 60820535 | NTSR1 |  | GG | 421 | AG | 56 | AA | 1 | 0 | 0.54 |  |  |
| rs4334545 | 20 | 60823622 | NTSR1 |  | GG | 288 | AG | 163 | AA | 27 | 0 | 0.54 |  |  |
| rs6090453 | 20 | 60825807 | NTSR1 |  | CC | 243 | CG | 197 | GG | 37 | 1 | 0.74 |  |  |
| rs6089784 | 20 | 60870007 | NTSR1 |  | GG | 282 | AG | 172 | AA | 23 | 1 | 0.62 |  |  |
| rs12612207 | 2 | 11724667 | NTSR2 |  | GG | 214 | AG | 218 | AA | 46 | 0 | 0.37 |  |  |
| rs463911 | 5 | 65024181 | NLN |  | GG | 161 | AG | 244 | AA | 73 | 0 | 0.21 |  |  |
| rs2548774 | 5 | 65084635 | NLN |  | GG | 316 | AG | 150 | AA | 12 | 0 | 0.24 |  |  |
| rs40107 | 5 | 65090567 | NLN |  | AA | 225 | AG | 216 | GG | 37 | 0 | 0.13 |  |  |
| rs1309822 | 5 | 65116336 | NLN |  | AA | 134 | AG | 260 | GG | 83 | 1 | 0.02 |  |  |
| rs2591933 | 5 | 65126301 | NLN |  | GG | 257 | AG | 180 | AA | 41 | 0 | 0.24 |  |  |
| rs2561196 | 5 | 65141378 | NLN |  | AA | 138 | AG | 235 | GG | 105 | 0 | 0.79 | 10 |  |
| rs2254485 | 5 | 65143971 | NLN |  | GG | 139 | AG | 234 | AA | 105 | 0 | 0.73 | 10 | Y |
| rs895379 | 5 | 65146545 | NLN |  | AA | 197 | AG | 218 | GG | 63 | 0 | 0.83 |  |  |
| rs2250861 | 5 | 65150759 | NLN |  | GG | 169 | AG | 237 | AA | 71 | 1 | 0.41 |  |  |
| rs16894446 | 5 | 65173217 | NLN |  | GG | 188 | AG | 223 | AA | 67 | 0 | 0.95 |  |  |

Note: Chr = Chromosome, Maj= major homozygote, N= number of subjects, Het=heterozygote, Min=minor homozygote, Miss = number of subjects missing genotype

* Hardy-Weinberg equilibrium was not tested on DRD4 VNTR because these 3 groups are combination of several genotypes

^ξ^ SNPs with the same numbers are clusters with high LD (r^2^>0.8), “Y” in “deleted” column means this SNP was deleted because of high LD
