# Supplementary material for: Contributions of Dopamine-Related Genes and Environmental Factors to Highly Sensitive Personality: A Multi-Step Neuronal System-Level Approach
Source: PLoS One. 2011 Jul 13;6(7):e21636. doi: 10.1371/journal.pone.0021636 (PMC3135587; doi:10.1371/journal.pone.0021636)
Supplement: Table S2 — Means and standard deviations of HSP score for each polymorphism, and main effects and post hoc comparisons of each locus. Rows in bold style were those that showed significant main effects and were used in subsequent multiple regression analysis. (DOC) [file pone.0021636.s002.doc]

Table S2. Means and standard deviations of HSP score for each polymorphism, and main effects and post hoc comparisons of each locus. Rows in bold style were those that showed significant main effects and were used in subsequent multiple regression analysis

| SNP | subsystem | deleted | Maj | Mean | SD | N | Het | Mean | SD | N | Min | Mean | SD | N | F | p | mh | mm | hm^§^ |
| --- | --- | --- | --- | --- | --- | --- | --- | --- | --- | --- | --- | --- | --- | --- | --- | --- | --- | --- | --- |
| **rs3842748** | Synthesis |  | **GG** | **121.96** | **15.63** | **447** | **CG** | **127.71** | **16.72** | **31** |  |  |  |  | **3.88** | **0.05** | **1** |  |  |
| rs2070762 |  |  | AA | 121.87 | 16.08 | 175 | AG | 122.55 | 15.97 | 219 | GG | 123.07 | 14.50 | 81 | 0.18 | 0.83 | 0 | 0 | 0 |
| rs6356 |  |  | AA | 121.93 | 16.21 | 325 | AG | 123.45 | 14.67 | 132 | GG | 121.57 | 15.47 | 21 | 0.46 | 0.63 | 0 | 0 | 0 |
| rs4930046 |  |  | AA | 122.45 | 16.23 | 368 | AG | 122.08 | 13.55 | 88 | GG | 120.76 | 16.50 | 21 | 0.13 | 0.88 | 0 | 0 | 0 |
| **rs4929966** |  |  | **GG** | **121.89** | **15.65** | **444** | **CG** | **128.12** | **16.10** | **34** |  |  |  |  | **4.98** | **0.03** | **1** |  |  |
| rs11238131 |  |  | AA | 123.14 | 15.20 | 131 | AG | 122.11 | 16.08 | 243 | GG | 121.78 | 15.82 | 103 | 0.26 | 0.77 | 0 | 0 | 0 |
| rs11238133 |  | Y | AA | 121.65 | 16.98 | 132 | AC | 123.18 | 14.99 | 224 | CC | 121.60 | 15.82 | 121 | 0.58 | 0.56 | 0 | 0 | 0 |
| rs11238134 |  |  | AA | 121.57 | 16.94 | 133 | AC | 123.16 | 15.02 | 223 | CC | 121.60 | 15.82 | 121 | 0.59 | 0.56 | 0 | 0 | 0 |
| rs3887825 |  |  | AA | 121.91 | 17.02 | 174 | AG | 122.99 | 14.08 | 221 | GG | 121.49 | 17.25 | 83 | 0.37 | 0.69 | 0 | 0 | 0 |
| rs3807566 |  |  | CC | 122.02 | 16.06 | 217 | AC | 123.38 | 14.42 | 204 | AA | 119.79 | 18.77 | 57 | 1.24 | 0.29 | 0 | 0 | 0 |
| rs1817074 |  |  | AA | 122.05 | 15.97 | 317 | AG | 122.92 | 15.11 | 141 | GG | 122.70 | 17.34 | 20 | 0.15 | 0.86 | 0 | 0 | 0 |
| rs7808025 |  | Y | GG | 122.26 | 15.87 | 316 | AG | 122.45 | 15.40 | 143 | AA | 122.63 | 17.14 | 19 | 0.01 | 0.99 | 0 | 0 | 0 |
| rs7786398 |  |  | GG | 121.94 | 17.00 | 141 | AG | 123.46 | 14.45 | 224 | AA | 120.58 | 16.53 | 113 | 1.32 | 0.27 | 0 | 0 | 0 |
| rs10499695 |  | Y | AA | 122.41 | 16.74 | 158 | AG | 123.00 | 14.37 | 225 | GG | 120.63 | 17.17 | 95 | 0.76 | 0.47 | 0 | 0 | 0 |
| rs6962356 |  |  | AA | 121.20 | 16.50 | 94 | AG | 122.45 | 15.75 | 359 | GG | 125.71 | 12.49 | 24 | 0.80 | 0.45 | 0 | 0 | 0 |
| rs6969081 |  |  | TT | 122.79 | 16.22 | 155 | AT | 122.73 | 15.18 | 233 | AA | 120.52 | 16.41 | 90 | 0.73 | 0.48 | 0 | 0 | 0 |
| rs2007153 |  |  | GG | 124.07 | 15.05 | 203 | AG | 121.25 | 15.56 | 204 | AA | 120.48 | 17.85 | 71 | 2.23 | 0.11 | 0 | 0 | 0 |
| rs1108580 |  |  | AA | 123.25 | 15.51 | 328 | AG | 121.01 | 15.61 | 134 | GG | 114.69 | 19.67 | 16 | 2.93 | 0.05 | 0 | 1 | 0 |
| **rs1611123** |  |  | **GG** | **123.50** | **15.21** | **332** | **AG** | **119.82** | **16.39** | **129** | **AA** | **118.65** | **19.07** | **17** | **3.04** | **0.05** | **1** | **0** | **0** |
| rs1541332 |  |  | AA | 122.39 | 15.88 | 447 | AG | 121.55 | 13.92 | 31 |  |  |  |  | 0.08 | 0.77 | 0 |  |  |
| rs2519154 |  |  | GG | 122.62 | 15.49 | 369 | AG | 121.24 | 16.64 | 108 |  |  |  |  | 0.64 | 0.43 | 0 |  |  |
| rs6479643 |  |  | GG | 122.96 | 15.80 | 242 | CG | 122.40 | 15.27 | 198 | CC | 118.19 | 17.75 | 37 | 1.48 | 0.23 | 0 | 0 | 0 |
| rs77905 |  |  | GG | 122.67 | 15.80 | 395 | AG | 120.72 | 15.49 | 83 |  |  |  |  | 1.05 | 0.31 | 0 |  |  |
| rs732833 |  |  | GG | 122.12 | 15.96 | 145 | AG | 123.14 | 14.70 | 235 | AA | 120.71 | 17.78 | 98 | 0.84 | 0.43 | 0 | 0 | 0 |
| rs2073837 |  |  | GG | 121.98 | 16.58 | 136 | AG | 123.02 | 15.81 | 231 | AA | 121.35 | 14.62 | 111 | 0.47 | 0.63 | 0 | 0 | 0 |
| rs737866 | Degradation  /Transport |  | AA | 122.51 | 15.88 | 246 | AG | 122.22 | 15.93 | 197 | GG | 121.74 | 14.16 | 35 | 0.04 | 0.96 | 0 | 0 | 0 |
| rs5993883 |  |  | AA | 122.73 | 15.48 | 172 | AC | 121.95 | 16.63 | 234 | CC | 122.65 | 13.43 | 72 | 0.14 | 0.87 | 0 | 0 | 0 |
| rs740603 |  |  | AA | 122.24 | 15.51 | 153 | AG | 122.59 | 16.31 | 240 | GG | 121.58 | 14.69 | 71 | 0.11 | 0.89 | 0 | 0 | 0 |
| rs2239393 |  | Y | AA | 122.07 | 15.69 | 198 | AG | 122.61 | 16.19 | 218 | GG | 122.21 | 14.55 | 62 | 0.07 | 0.94 | 0 | 0 | 0 |
| rs4680 |  |  | VV | 122.93 | 15.70 | 256 | VM | 122.03 | 16.11 | 178 | MM | 120.10 | 15.42 | 30 | 0.50 | 0.60 | 0 | 0 | 0 |
| rs4646316 |  |  | GG | 122.36 | 16.11 | 209 | AG | 122.07 | 15.81 | 210 | AA | 123.50 | 14.32 | 58 | 0.19 | 0.83 | 0 | 0 | 0 |
| rs165774 |  |  | GG | 121.95 | 15.46 | 369 | AG | 123.64 | 16.70 | 109 |  |  |  |  | 0.97 | 0.32 | 0 |  |  |
| rs929095 |  |  | GG | 120.77 | 15.67 | 196 | CG | 123.43 | 15.74 | 282 |  |  |  |  | 3.31 | 0.07 | 0 |  |  |
| rs1181286 |  |  | CC | 122.33 | 15.64 | 396 | AC | 122.35 | 16.35 | 82 |  |  |  |  | 0.00 | 0.99 | 0 |  |  |
| rs1181289 |  | Y | TT | 122.33 | 15.64 | 396 | AT | 122.35 | 16.35 | 82 |  |  |  |  | 0.00 | 0.99 | 0 |  |  |
| rs5906974 |  | Y | GG | 122.21 | 15.39 | 217 | AG | 122.44 | 16.07 | 261 |  |  |  |  | 0.02 | 0.88 | 0 |  |  |
| rs909525 |  |  | GG | 122.21 | 15.39 | 217 | AG | 122.52 | 16.04 | 260 |  |  |  |  | 0.05 | 0.83 | 0 |  |  |
| MAOA VNTR |  |  | VV | 122.20 | 15.43 | 210 | VM | 122.15 | 15.97 | 259 |  |  |  |  | 0.00 | 0.97 | 0 |  |  |
| rs1799836 |  |  | AA | 121.61 | 15.53 | 352 | AG | 124.37 | 16.24 | 126 |  |  |  |  | 2.88 | 0.09 | 0 |  |  |
| rs6651806 |  |  | AA | 121.98 | 15.79 | 381 | AC | 123.74 | 15.57 | 97 |  |  |  |  | 0.97 | 0.32 | 0 |  |  |
| rs5905512 |  |  | AA | 121.29 | 15.53 | 284 | AG | 123.87 | 15.98 | 194 |  |  |  |  | 3.12 | 0.08 | 0 |  |  |
| rs1042098 |  |  | AA | 122.01 | 15.76 | 383 | AG | 123.61 | 15.82 | 94 |  |  |  |  | 0.77 | 0.38 | 0 |  |  |
| rs40184 |  |  | GG | 122.10 | 15.92 | 260 | AG | 123.10 | 15.34 | 188 | AA | 119.53 | 16.97 | 30 | 0.72 | 0.49 | 0 | 0 | 0 |
| rs6347 |  |  | AA | 122.62 | 16.04 | 370 | AG | 121.35 | 14.73 | 108 |  |  |  |  | 0.54 | 0.46 | 0 |  |  |
| rs37022 |  |  | AA | 122.17 | 15.77 | 120 | AT | 122.24 | 16.11 | 239 | TT | 122.69 | 15.11 | 119 | 0.04 | 0.96 | 0 | 0 | 0 |
| **rs2975292** |  |  | **GG** | **121.94** | **15.55** | **371** | **CG** | **125.37** | **16.22** | **95** | **CC** | **112.09** | **11.20** | **11** | **4.28** | **0.01** | **0** | **1** | **1** |
| rs10053602 |  | Y | AA | 121.94 | 15.61 | 373 | AG | 125.02 | 16.28 | 93 | GG | 113.83 | 12.27 | 12 | 3.25 | 0.04 | 0 | 0 | 1 |
| rs403636 |  |  | CC | 122.32 | 16.52 | 222 | AC | 122.43 | 15.37 | 204 | AA | 122.04 | 14.00 | 52 | 0.01 | 0.99 | 0 | 0 | 0 |
| rs2937639 |  |  | AA | 122.11 | 15.32 | 350 | AG | 123.68 | 16.66 | 114 | GG | 116.93 | 18.34 | 14 | 1.28 | 0.28 | 0 | 0 | 0 |
| rs686 | Receptor | Y | AA | 122.40 | 15.89 | 351 | AG | 122.75 | 14.76 | 114 | GG | 116.85 | 20.28 | 13 | 0.83 | 0.44 | 0 | 0 | 0 |
| rs1799914 |  |  | GG | 122.55 | 15.75 | 442 | AG | 119.64 | 15.74 | 36 |  |  |  |  | 1.14 | 0.29 | 0 |  |  |
| rs4532 |  | Y | AA | 122.42 | 15.88 | 351 | AG | 122.87 | 14.79 | 114 | GG | 115.50 | 20.56 | 12 | 1.20 | 0.30 | 0 | 0 | 0 |
| rs5326 |  |  | GG | 122.45 | 15.93 | 296 | AG | 121.90 | 16.04 | 157 | AA | 124.52 | 10.94 | 23 | 0.29 | 0.75 | 0 | 0 | 0 |
| rs265981 |  |  | GG | 122.38 | 15.84 | 356 | AG | 122.92 | 14.85 | 110 | AA | 115.50 | 20.56 | 12 | 1.21 | 0.30 | 0 | 0 | 0 |
| rs6277 |  | Y | GG | 122.52 | 15.89 | 408 | AG | 121.24 | 14.97 | 70 |  |  |  |  | 0.39 | 0.53 | 0 |  |  |
| rs2734839 |  |  | GG | 122.53 | 15.82 | 398 | AG | 120.80 | 15.19 | 64 |  |  |  |  | 0.67 | 0.41 | 0 |  |  |
| rs17529477 |  |  | GG | 122.59 | 16.05 | 435 | AG | 119.79 | 12.19 | 43 |  |  |  |  | 1.23 | 0.27 | 0 |  |  |
| rs17601612 |  |  | GG | 122.61 | 15.99 | 441 | CG | 119.08 | 12.24 | 37 |  |  |  |  | 1.71 | 0.19 | 0 |  |  |
| rs4245147 |  |  | AA | 122.36 | 16.01 | 322 | AG | 122.00 | 15.18 | 139 | GG | 124.53 | 16.14 | 17 | 0.20 | 0.82 | 0 | 0 | 0 |
| **rs7131056** |  |  | **CC** | **118.77** | **16.48** | **156** | **AC** | **122.67** | **15.26** | **233** | **AA** | **127.70** | **14.17** | **89** | **9.54** | **<0.01** | **1** | **1** | **1** |
| rs2134655 |  |  | GG | 121.96 | 16.39 | 252 | AG | 123.23 | 14.94 | 198 | AA | 120.30 | 15.03 | 27 | 0.61 | 0.54 | 0 | 0 | 0 |
| rs324035 |  |  | CC | 122.58 | 15.43 | 343 | AC | 121.74 | 16.55 | 120 | AA | 121.47 | 17.49 | 15 | 0.15 | 0.86 | 0 | 0 | 0 |
| rs10934256 |  |  | CC | 122.98 | 16.64 | 256 | AC | 122.09 | 14.38 | 196 | AA | 117.04 | 16.28 | 25 | 1.66 | 0.19 | 0 | 0 | 0 |
| rs7638961 |  |  | AA | 122.97 | 16.63 | 228 | AG | 122.49 | 14.66 | 212 | GG | 117.63 | 15.80 | 38 | 1.90 | 0.15 | 0 | 0 | 0 |
| DRD4VNTR |  |  | 4/4 | 121.28 | 16.29 | 279 | 2/+ | 123.93 | 14.25 | 140 | other | 123.39 | 16.65 | 57 | 1.46 | 0.23 | 0 | 0 | 0 |
| rs7933153 |  |  | CC | 122.31 | 15.83 | 452 | AC | 122.85 | 14.64 | 26 |  |  |  |  | 0.03 | 0.87 | 0 |  |  |
| rs12720366 |  |  | AA | 122.87 | 15.82 | 247 | AG | 122.09 | 14.84 | 191 | GG | 120.23 | 19.38 | 40 | 0.52 | 0.59 | 0 | 0 | 0 |
| rs12720424 |  |  | AA | 121.93 | 16.04 | 268 | AC | 122.85 | 15.67 | 181 | CC | 122.86 | 13.78 | 29 | 0.20 | 0.82 | 0 | 0 | 0 |
| rs11604855 |  |  | GG | 122.06 | 15.37 | 306 | AG | 123.13 | 16.71 | 155 | AA | 120.00 | 13.88 | 17 | 0.43 | 0.65 | 0 | 0 | 0 |
| rs12233771 |  |  | TT | 122.72 | 17.60 | 120 | AT | 122.56 | 15.13 | 247 | AA | 121.41 | 15.08 | 111 | 0.25 | 0.78 | 0 | 0 | 0 |
| rs9884669 |  |  | CC | 121.82 | 16.39 | 228 | AC | 122.70 | 14.92 | 214 | AA | 123.39 | 16.70 | 36 | 0.26 | 0.77 | 0 | 0 | 0 |
| rs4102942 |  |  | GG | 121.34 | 15.16 | 105 | AG | 122.76 | 15.50 | 325 | AA | 121.53 | 18.86 | 47 | 0.38 | 0.68 | 0 | 0 | 0 |
| rs7655090 |  |  | AA | 121.21 | 15.62 | 133 | AG | 123.19 | 16.21 | 239 | GG | 121.82 | 14.86 | 106 | 0.75 | 0.48 | 0 | 0 | 0 |
| rs10033951 |  |  | GG | 120.64 | 15.35 | 92 | AG | 122.52 | 15.84 | 328 | AA | 123.98 | 15.88 | 58 | 0.87 | 0.42 | 0 | 0 | 0 |
| rs2867383 |  |  | AA | 123.14 | 15.84 | 145 | AG | 122.22 | 15.96 | 232 | GG | 121.46 | 15.23 | 101 | 0.35 | 0.70 | 0 | 0 | 0 |
| rs1850744 |  |  | GG | 122.86 | 16.18 | 303 | AG | 121.48 | 15.01 | 174 |  |  |  |  | 0.85 | 0.36 | 0 |  |  |
| rs7685396 |  |  | CC | 122.47 | 15.22 | 319 | AC | 121.88 | 16.99 | 152 |  |  |  |  | 0.14 | 0.71 | 0 |  |  |
| rs4697695 |  |  | GG | 122.68 | 15.41 | 276 | AG | 121.98 | 16.03 | 173 | AA | 121.85 | 17.10 | 26 | 0.12 | 0.89 | 0 | 0 | 0 |
| rs2405254 | Modulation |  | TT | 122.75 | 15.62 | 138 | AT | 122.81 | 16.68 | 216 | AA | 121.05 | 14.19 | 124 | 0.56 | 0.57 | 0 | 0 | 0 |
| rs12313658 |  |  | GG | 122.30 | 15.53 | 438 | AG | 122.68 | 18.20 | 40 |  |  |  |  | 0.02 | 0.89 | 0 |  |  |
| rs1024076 |  |  | GG | 122.57 | 15.91 | 222 | AG | 123.11 | 16.02 | 210 | AA | 117.65 | 12.99 | 46 | 2.33 | 0.10 | 0 | 0 | 1 |
| rs10506933 |  |  | CC | 121.72 | 14.97 | 198 | CG | 123.57 | 16.06 | 223 | GG | 119.67 | 16.94 | 57 | 1.66 | 0.19 | 0 | 0 | 0 |
| rs2427399 |  |  | GG | 122.39 | 16.80 | 287 | AG | 122.19 | 14.52 | 167 | AA | 122.67 | 10.63 | 24 | 0.01 | 0.99 | 0 | 0 | 0 |
| **rs6062460** |  |  | **GG** | **122.87** | **15.78** | **421** | **AG** | **118.40** | **15.06** | **57** |  |  |  |  | **4.06** | **0.04** | **1** |  |  |
| rs4334545 |  |  | GG | 122.41 | 16.58 | 288 | AG | 122.37 | 14.62 | 163 | AA | 121.30 | 13.52 | 27 | 0.06 | 0.94 | 0 | 0 | 0 |
| rs6090453 |  |  | CC | 123.39 | 16.65 | 243 | CG | 121.95 | 14.48 | 197 | GG | 117.59 | 15.79 | 37 | 2.28 | 0.10 | 0 | 1 | 0 |
| rs6089784 |  |  | GG | 122.94 | 16.22 | 282 | AG | 121.26 | 15.38 | 172 | AA | 123.39 | 12.67 | 23 | 0.66 | 0.52 | 0 | 0 | 0 |
| **rs12612207** |  |  | **GG** | **123.76** | **15.48** | **214** | **AG** | **122.06** | **15.76** | **218** | **AA** | **117.00** | **16.06** | **46** | **3.58** | **0.03** | **0** | **1** | **1** |
| rs463911 |  |  | GG | 120.61 | 15.04 | 161 | AG | 123.93 | 16.16 | 244 | AA | 120.79 | 15.52 | 73 | 2.58 | 0.08 | 1 | 0 | 0 |
| rs2548774 |  |  | GG | 121.73 | 15.87 | 316 | AG | 123.64 | 15.79 | 150 | AA | 121.92 | 11.56 | 12 | 0.75 | 0.47 | 0 | 0 | 0 |
| rs40107 |  |  | AA | 120.66 | 15.94 | 225 | AG | 123.83 | 16.04 | 216 | GG | 123.78 | 11.69 | 37 | 2.42 | 0.09 | 1 | 0 | 0 |
| rs1309822 |  |  | AA | 120.68 | 14.98 | 134 | AG | 122.98 | 16.58 | 260 | GG | 122.94 | 14.31 | 83 | 1.02 | 0.36 | 0 | 0 | 0 |
| rs2591933 |  |  | GG | 121.49 | 15.85 | 257 | AG | 122.31 | 15.83 | 180 | AA | 127.73 | 13.89 | 41 | 2.79 | 0.06 | 0 | 1 | 1 |
| **rs2561196** |  |  | **AA** | **124.83** | **16.35** | **138** | **AG** | **122.11** | **15.45** | **235** | **GG** | **119.55** | **15.25** | **105** | **3.42** | **0.03** | **0** | **1** | **0** |
| rs2254485 |  | Y | GG | 124.81 | 16.29 | 139 | AG | 122.12 | 15.48 | 234 | AA | 119.55 | 15.25 | 105 | 3.41 | 0.03 | 0 | 1 | 0 |
| **rs895379** |  |  | **AA** | **119.05** | **15.63** | **197** | **AG** | **124.31** | **15.32** | **218** | **GG** | **125.78** | **15.96** | **63** | **7.73** | **<0.01** | **1** | **1** | **0** |
| rs2250861 |  |  | GG | 121.62 | 15.34 | 169 | AG | 121.84 | 16.06 | 237 | AA | 125.51 | 15.53 | 71 | 1.74 | 0.18 | 0 | 0 | 0 |
| **rs16894446** |  |  | **GG** | **124.78** | **15.77** | **188** | **AG** | **121.43** | **15.71** | **223** | **AA** | **118.49** | **14.91** | **67** | **4.71** | **0.01** | **1** | **1** | **0** |

^§^Results of Post-Hoc comparison. mh= Maj vs Het, mm= Maj vs Min, hm=Het vs Min, 1 means significant difference between groups
