# Supplementary material for: Contributions of Dopamine-Related Genes and Environmental Factors to Highly Sensitive Personality: A Multi-Step Neuronal System-Level Approach
Source: PLoS One. 2011 Jul 13;6(7):e21636. doi: 10.1371/journal.pone.0021636 (PMC3135587; doi:10.1371/journal.pone.0021636)
Supplement: Supporting Information S1 — English and Chinese versions of scales used in this study. (DOC) [file pone.0021636.s003.doc]

**Highly Sensitive Person Scale**

高敏感性人格问卷

Instructions: Answer each of the following questions according to the way you personally feel, using the following scale:

1 2 3 4 5 6 7

**Not at all Moderately Extremely**

指导语：请根据你的感受回答下列问题。答题时请使用以下的标准：

1 2 3 4 5 6 7

**一点都不 中等程度 极端地**

1. Are you easily overwhelmed by strong sensory input?

你容易受不了强烈的感觉刺激吗？

1. Do you seem to be aware of subtleties in your environment?

你对周围环境中的细节似乎特别留心吗？

1. Do other people's moods affect you?

别人的情绪容易影响你吗？

1. Do you tend to be very sensitive to pain?

你的痛觉很敏感吗？

1. Do you find yourself needing to withdraw during busy days, into bed or into a darkened room or any place where you can have some privacy and relief from stimulation?

你觉得自己忙碌的时候需要躲起来吗？或者在你激动后，会躲进被窝里，躲到漆黑的房间，或是躲到任何一个能够使你独处不受干扰并能得到舒缓的地方吗？

1. Are you particularly sensitive to the effects of caffeine?

你对咖啡因特别敏感吗？

1. Are you easily overwhelmed by things like bright lights, strong smells, coarse fabrics, or sirens close by?

你容易受不了像强光、强烈的气味、粗糙的布料或周围的警铃等事物吗？

1. Do you have a rich, complex inner life?

你有丰富而复杂的内心世界吗？

1. Are you made uncomfortable by loud noises?

你会因为喧闹的噪声而觉得不舒服吗？

1. Are you deeply moved by the arts or music?

你会被艺术或音乐深深感动吗？

1. Does your nervous system sometimes feel so frazzled that you just have to get away by yourself?

你有时候会感到非常疲惫以至于你只得一个人逃避吗？

1. Are you conscientious?

你很认真负责吗？

1. Do you startle easily?

你很容易受到惊吓吗？

1. Do you get rattled when you have a lot to do in a short amount of time?

如果在短时间内你必须做许多事情，你会感到慌乱吗？

1. When people are uncomfortable in a physical environment, do you tend to know what needs to be done to make it more comfortable (like changing the lighting or the seating)?

当人们因周围环境而感到不舒服时，你往往知道要怎样做才能使环境变得更舒服些吗（例如改变灯光或者调整座位）？

1. Are you annoyed when people try to get you to do too many things at once?

如果人们试图让你同时做太多件事情，你会觉得很厌烦吗？

1. Do you try hard to avoid making mistakes or forgetting things?

你总是很努力地避免犯错、避免忘记事情吗？

1. Do you make a point to avoid violent movies and TV shows?

你尽量不看有暴力内容的电影和电视节目吗？

1. Do you become unpleasantly aroused when a lot is going on around you?

当你身边同时发生很多事的时候，你会感到不舒服吗？

1. Does being very hungry create a strong reaction in you, disrupting your concentration or mood?

你在很饿的时候会有强烈的反应，以至于影响你的注意力和情绪吗？

1. Do changes in your life shake you up?

生活中的变动会给你带来冲击吗？

1. Do you notice and enjoy delicate or fine scents, tastes, sounds, works of art?

你很留意并享受精致或美妙的气味、味道、声音及艺术品吗？

1. Do you find it unpleasant to have a lot going on at once?

同时做很多事情会令你感到不快吗？

1. Do you make it a high priority to arrange your life to avoid upsetting or overwhelming situations?

你必须要事先安排好你的生活，以避免出现使你心烦意乱或者令你无力抵抗的情况吗？

1. Are you bothered by intense stimuli, like loud noises or chaotic scenes?

高强度的刺激，如喧闹的噪音或混乱的景象会令你烦扰吗？

1. When you must compete or be observed while performing a task, do you become so nervous or shaky that you do much worse than you would otherwise?

当你必须与他人竞争时，或者你做事的时候有人观察时，你会感到很紧张、情绪不稳定以至于表现得比平常差吗？

1. When you were a child, did your parents or teachers seem to see you as sensitive or shy?

在你小的时候，你的父母或老师似乎都认为你是一个敏感或害羞的孩子吗？

Source of the English version: Aron EN, Aron A (1997) Sensory-processing sensitivity and its relation to introversion and emotionality. J Pers Soc Psychol 73:345-368

**Parental Warmth and Acceptance Scale**

父母温暖和接受量表

**Please indicate whether you agree or disagree with the following statements.**

1 2 3 4 5 6

Strongly Disagree Slightly Slightly Agree Strongly

Disagree Disagree Agree Agree

下面有一些关于你父母的问题。你在多大程度上同意下列陈述？

1＝完全不同意

2＝不同意

3＝有点不同意

4＝有点同意

5＝同意

6＝完全同意

|  | 完全  不同意 | 不同意 | 有点  不同意 | 有点  同意 | 同意 | 完全  同意 |
| --- | --- | --- | --- | --- | --- | --- |
| 1. My parents really understand me   父母确实理解我 | 1 | 2 | 3 | 4 | 5 | 6 |
| 1. My parents make me feel that I am a burden to them   父母使我觉得自己是他们的负担 | 1 | 2 | 3 | 4 | 5 | 6 |
| 1. I find it hard to please my parents   我发现很难使父母高兴 | 1 | 2 | 3 | 4 | 5 | 6 |
| 1. I don’t really feel that my parents love me   我没有真正感到父母爱我 | 1 | 2 | 3 | 4 | 5 | 6 |
| 1. I know that my parents will “be there” for me if I need them   我知道，当我需要的时候，父母会帮助我 | 1 | 2 | 3 | 4 | 5 | 6 |
| 1. My parents don’t pay attention when I talk about things that are important to me   当我谈论对我很重要的事情时，父母并不太注意 | 1 | 2 | 3 | 4 | 5 | 6 |
| 1. My parents really enjoy spending time with me   父母确实喜欢与我在一起 | 1 | 2 | 3 | 4 | 5 | 6 |
| 1. My parents let me know through their words or actions that they love me   父母通过语言加行动，让我知道他们爱我 | 1 | 2 | 3 | 4 | 5 | 6 |
| 1. My parents and I often go places and do enjoyable things together   我和父母经常一起出去做一些有趣的事 | 1 | 2 | 3 | 4 | 5 | 6 |
| 1. My parents seldom help me figure out or fix things   父母很少帮助我出点子和解决问题 | 1 | 2 | 3 | 4 | 5 | 6 |
| 1. I don’t share my secrets and private feelings with my parents   我不与父母分享我的秘密和个人感情 | 1 | 2 | 3 | 4 | 5 | 6 |

Reverse-scored items: 2, 3, 4, 6, 10, and 11

反向记分题 2 3 4 6 10 11

Source of both English and Chinese versions: Greenberger, E., Chen, C., Tally, S., & Dong, Q. (2000). Family, peer, and individual correlates of depressive symptomatology among U.S. and Chinese adolescents. *Journal of Consulting and Clinical Psychology, 68*, 209-219.

.

**Stressful Life Events**

生活压力事件量表

**People your age sometimes experience stressful events or big changes. Among the following list of things, what has happened to you? (Note: Circle “no” if the question is not applicable to you)**

和你同龄的人有时会经历许多对生活有压力的事件或生活上有重大变化。

对你来说下面所列举的事件哪些发生了（如问题不适合你，请选择“否”）。

|  | Primary school or before  小学及以前 | | Secondary school years  中学期间 | | College years  大学期间 | |
| --- | --- | --- | --- | --- | --- | --- |
|  | 是  Yes | 否  No | 是  Yes | 否  No | 是  Yes | 否  No |
| - - 1. I moved to a new school我转入新的学校 |  |  |  |  |  |  |
| - - 1. I broke up with my boyfriend or girlfriend我与我的男（女）朋友分手了 |  |  |  |  |  |  |
| - - 1. A close friend moved quite far away一个好朋友搬到很远的地方去了 |  |  |  |  |  |  |
| - - 1. One of my close friends died我的好朋友中有一个死了 |  |  |  |  |  |  |
| - - 1. One of my close friends became seriously ill or was injured我的好朋友中有一个得了重病或受到了伤害 |  |  |  |  |  |  |
| - - 1. One of my parents became seriously ill, or was injured, or was hospitalized我父母中有一个得了重病，或受了伤，或住了院 |  |  |  |  |  |  |
| - - 1. My mother or father died我母亲或父亲过世了 |  |  |  |  |  |  |
| - - 1. A brother or sister died我的一个兄弟姐妹过世了 |  |  |  |  |  |  |
| - - 1. One of my grandparents died我祖父母中有一个过世了 |  |  |  |  |  |  |
| - - 1. One of my parents got or was put in a job worse than the previous one我父母中有一个换到或被调到一个比以前差的工作 |  |  |  |  |  |  |
| - - 1. I did not get into a club or sports team I really wanted to be involved in 我不能参加我其实很想参加的俱乐部或体育运动队 |  |  |  |  |  |  |
| - - 1. Had a serious “falling-out” or ended a friendship with a close friend我和好朋友大吵了一顿或中止了关系 |  |  |  |  |  |  |
| - - 1. One of my parents lost his/her job我父母中有一个失业了 |  |  |  |  |  |  |
| - - 1. My Parents were divorced or separated我父母离婚或分居了 |  |  |  |  |  |  |
| - - 1. My family’s financial situation became a worry我们家的经济状况变将令人担忧 |  |  |  |  |  |  |
| - - 1. I became seriously ill, or was injured, or was hospitalized我得了重病，或受了伤，或住了院 |  |  |  |  |  |  |
| - - 1. A brother or sister became seriously ill, or was injured, or was hospitalized我的一个兄弟或姐妹得了重病，或受了伤，或住了院 |  |  |  |  |  |  |
| - - 1. My grades in school went down a lot我的学业成绩下降了很多 |  |  |  |  |  |  |
| - - 1. I flunked a course我考试有一门不及格 |  |  |  |  |  |  |
| - - 1. I was suspended or expelled from school我被学校停学或开除了 |  |  |  |  |  |  |
| - - 1. I was assaulted, robbed, or the victim of other violent crime我被攻击、抢劫或成为其它暴力犯罪的受害者 |  |  |  |  |  |  |
| - - 1. My parents argued much more with each other我父母吵架比以前多了许多 |  |  |  |  |  |  |
| - - 1. A close family member was arrested一个近亲被逮捕了 |  |  |  |  |  |  |
| - - 1. My physical appearance got worse (weight, acne, etc.) 我的貌体变得越来越糟糕（如体重、粉刺等） |  |  |  |  |  |  |

Adapted from several sources, with the closest version in both English and Chinese from: Greenberger, E., Chen, C., Tally, S., & Dong, Q. (2000). Family, peer, and individual correlates of depressive symptomatology among U.S. and Chinese adolescents. *Journal of Consulting and Clinical Psychology, 68*, 209-219.

*.*
